# Impact of drought associated with high temperatures on *Coffea canephora* plantations: a case study in Espírito Santo State, Brazil

**DOI:** 10.1038/s41598-020-76713-y

**Published:** 2020-11-12

**Authors:** Luan Peroni Venancio, Roberto Filgueiras, Everardo Chartuni Mantovani, Cibele Hummel do Amaral, Fernando França da Cunha, Francisco Charles dos Santos Silva, Daniel Althoff, Robson Argolo dos Santos, Paulo Cezar Cavatte

**Affiliations:** 1 Agricultural Engineering Department, Federal University of Viçosa (UFV), Viçosa, 36570-900 Brazil; 2 Forest Engineering Department, Federal University of Viçosa (UFV), Viçosa, 36570-900 Brazil; 3 Biology Department, Federal University of Espírito Santo (UFES), Alegre, 29500-000 Brazil

**Keywords:** Climate change, Climate-change impacts, Socioeconomic scenarios, Climate sciences, Environmental sciences, Hydrology, Natural hazards

## Abstract

Droughts are major natural disasters that affect many parts of the world all years and recently affected one of the major conilon coffee-producing regions of the world in state of Espírito Santo, which caused a huge crisis in the sector. Therefore, the objective of this study was to conduct an analysis with technical-scientific basis of the real impact of drought associated with high temperatures and irradiances on the conilon coffee (*Coffea canephora* Pierre ex Froehner) plantations located in the north, northwest, and northeast regions of the state of Espírito Santo, Brazil. Data from 2010 to 2016 of rainfall, air temperature, production, yield, planted area and surface remote sensing were obtained from different sources, statistically analyzed, and correlated. The 2015/2016 season was the most affected by the drought and high temperatures (mean annual above 26 °C) because, in addition to the adverse weather conditions, coffee plants were already damaged by the climatic conditions of the previous season. The increase in air temperature has higher impact (negative) on production than the decrease in annual precipitation. The average annual air temperatures in the two harvest seasons that stood out for the lowest yields (i.e. 2012/2013 and 2015/2016) were approximately 1 °C higher than in the previous seasons. In addition, in the 2015/2016 season, the average annual air temperature was the highest in the entire series. The spatial and temporal distribution of Enhanced Vegetation Index values enabled the detection and perception of droughts in the conilon coffee-producing regions of Espírito Santo. The rainfall volume accumulated in the periods from September to December and from April to August are the ones that most affect coffee yield. The conilon coffee plantations in these regions are susceptible to new climate extremes, as they continue to be managed under irrigation and full sun. The adoption of agroforestry systems and construction of small reservoirs can be useful to alleviate these climate effects, reducing the risk of coffee production losses and contributing to the sustainability of crops in Espírito Santo.

## Introduction

Climate change is one of the most important concerns and challenges for scientists, managers, and decision makers throughout the world^1,2^. The agriculture appears to be one of the human activities most vulnerable to climate changes due to its large dependence on environmental conditions^2,3^. For coffee producers, climate change also is a huge challenge, since the drought is the main environmental restriction that affects coffee growth and production^4,5^, reducing the yield up to 80% in very dry years in some marginal regions with no irrigation^5^. In addition, coffee has been categorized as a highly sensitive plant species to progressive climate change^6^.

A recent study of Magrach et al.^7^ suggest that many currently *Coffea canephora* Pierre ex Froehner cultivated areas will become less suitable for cultivation with the impact of the climate change (projections of rising temperatures and altered precipitation patterns). According to the authors, this species could lose 55% of currently suitable areas, mostly within western Africa and southeast Brazil. This is very alarming because the Espírito Santo state, located in the southeast Brazil, is the largest producer of *Coffea canephora* in the country, responsible for 70% of its production and approximately 15% of the global production, despite of occupying only 0.55% of the Brazilian territory^8,9^. In economic terms, *C. canephora* is responsible for 35% of the gross domestic product (GDP) of Espírito Santo and for the generation of 250,000 direct and indirect jobs, which makes it considered the main agricultural product of the state^9^. Thus, the occurrence of climate-driven problems, such as drought and high temperatures, can threaten sustainability of that activity in this region, given its impacts on coffee production.

Previous studies on the effect of water deficit on growth, photosynthesis and carbon metabolism of coffee plants have provided important insights, specially, the susceptibility of these plants to climate change. Pinheiro et al.^10^ studied four genotypes of conilon coffee, two drought-tolerant and two sensitive genotypes, which were submitted to a slowly imposed water deficit. The authors verified that, regardless of the genotypes clones investigated, the net carbon assimilation rate decreased under drought stress. Thus, even for tolerant clones, the water deficit can affect the growth, photosynthesis and, consequently, yield. In a study with the same clones and same treatments used by the previous authors, Praxedes et al.^11^ verified that, regardless of the stress severity, decrease in reserve accumulation remarkably and, regardless of the clone evaluated, drought led to sharper decreases in stomatal conductance and, therefore, to photosynthetic capacity decrease. On the other hand, drought-tolerant genotypes can increase the long-term water use efficiency (WUE)^12^. In any case, greater WUE associated with decreases in stomatal conductance generally lead to lower rates of transpiration. However, this also results in less latent heat loss, potentially increasing leaf temperatures, which in turn can harm the crop performance in a global warming scenario^6^.

In the Espírito Santo state, the region of interest of this study, drought years are associated to low rainfall during the winter and fall seasons (from April to September). This period also matches the time of the coffee harvest, and droughts tend to compromise the crops vigor^13^. In general, drought periods can lead to plant death, while moderate drought periods are also very damaging to coffee growers by affecting flowering, bean development, and, consequently, coffee production^14^. Therefore, the use of irrigation is essential to guarantee adequate crop yields.

In most situations, drought impacts can be greatly aggravated by supra-optimal air temperatures^15^. Extreme temperatures, depending on their intensity, duration, and speed of imposition, impair cell metabolic processes (e.g., photosynthesis)^16,17^, growth and survival of plants, as well as their economic exploitation^16^. In the region of study, it is common the occurrence of summer temperatures reaching up to 40 °C. Exceeding this level during the phase of grain filling is critical and will lead to grains wilting and, consequently, significant decreases in the crops yield^13^. During blossoming, especially if associated with a prolonged dry season, high air temperature may cause abortion of flowers, directly impacting the production^17^. In general terms, high temperature are harmful for coffee production and, the attenuation of the incident solar radiation, the temperature, and the evaporative demand (e.g., using a agroforestry system) can result in better conditions for the maintenance of the gas exchanges with positive effects on the production, especially in marginal regions where coffee cultivation is characterized for suffering with water deficit associated with extreme temperatures and excess irradiance^13^.

Under drought conditions, agroforestry systems can bring several positive benefits to the coffee plantations, as they have been reported to mitigate the variability in microclimate^18–20^, and to promote increases soil moisture^21,22^ and in water infiltration in the soil^23^. Besides that, agroforestry systems help reduce the maximum air temperatures^19,24^ and the intensity of photosynthetically active radiation^19^. According to findings of Gidey et al.^25^, the coffee production under agroforestry systems has a higher level of resilience when facing future climate change and reinforces the idea of agroforestry systems help adapt to the negative impacts of climate change on the coffee production.

The state of Espírito Santo harvested the largest harvest in history in 2013/2014 season^26^, however in the two subsequent seasons the climatic conditions imposed significant limitations on the productive capacity of the crops, which harmed the regional coffee production and may be an indicative of what to expect from climate change in the near future. Despite the aforementioned studies, there is no information on the conilon coffee responses to drought and high temperature at the regional scale for the Espírito Santo state. This study, by integrating large datasets, is an effort to fill this gap. Thus, the objective of this study was to analyze the variation in the production and yield of the conilon coffee cultivated in the north, northeast, and northwest regions of Espírito Santo, Brazil, based on data of rainfall, air temperature, and satellite-based optical vegetation index. Specifically, the study aims to identify which period of the crop cycle is most sensitive to the stressful environmental conditions frequently seen in the region (water deficit associated with high temperatures and irradiances). This study also provides information for the decision-making related to the sustainability of the coffee sector, especially, in the Espírito Santo state, where Conilon coffee is considered the main agricultural product and may be jeopardized by climate change.

The subsequent sections of this manuscript are organized as follow: “Methods”, where we describe the (1) area of study, (2) data acquisition (monthly rainfall and air temperature, production, yield, planted area and satellite-based optical vegetation index) for the main conilon coffee producing municipalities in the Espírito Santo state, Brazil, (3) data processing and (4) statistical approaches used herein; the “Results and discussion” was divided in five subsections as (1) climatic conditions during the study period, (2) relationship between drop in production and climate conditions, (3) combined effect of rainfall and air temperature on coffee production, (4) identification of which period of the crop cycle is most affected by the rainfall volumes, and (4) spatial–temporal drought impacts on crop production using a satellite-based optical vegetation index; and, in “Conclusions”, we present the overall conclusions of this research.

## Methods

### Study area

**Figure 1:**
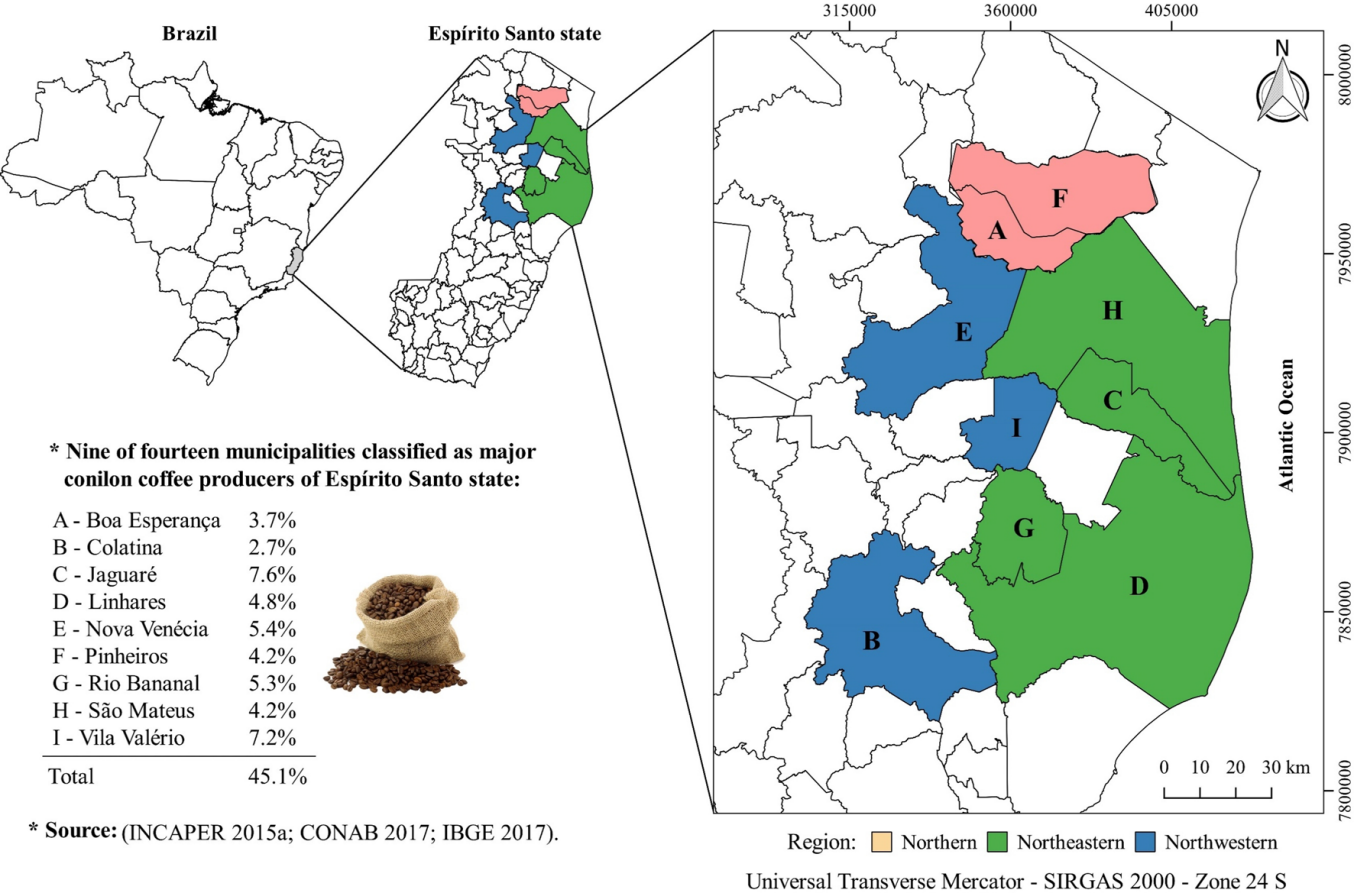
Geographical location of nine of the fourteen municipalities classified as major producers of *Coffea canephora* of the state of Espírito Santo and their respective average percentages of contribution to the state production.

The study area involves the municipalities of the northern (Boa Esperança and Pinheiros), northeastern (Jaguaré, Linhares, Rio Bananal, São Mateus) and northwestern (Colatina, Nova Venécia and Vila Valério) regions of the state of Espírito Santo, Brazil (Fig. 1). These nine municipalities are part of the fourteen classified as major producers of coffee conilon in this state^9^. The plantations of conilon coffee in these municipality are clonal, formed by genotypes of high production capacity, which are recommended by the Capixaba Institute of Research, Technical Assistance and Rural Extension (INCAPER).

One of the main characteristics of these municipalities is the flat relief, which enables the mechanization of many agricultural practices and the use of different irrigation systems. The irrigation systems commonly used are those of localized application (dripper and micro-sprinkler) and by sprinkler (center pivot and conventional sprinkler), and the source of water for this purpose is mainly rivers and small reservoirs^27^. It is important to point out, which in these municipalities more than 90% of coffee plantations are irrigated^9^ . Most coffee areas in these three regions are considered small and medium-sized properties^9^. The predominant soil is *Latossolo Vermelho-Amarelo* (Oxisol)^28^.

### Data acquisition

**Figure 2:**
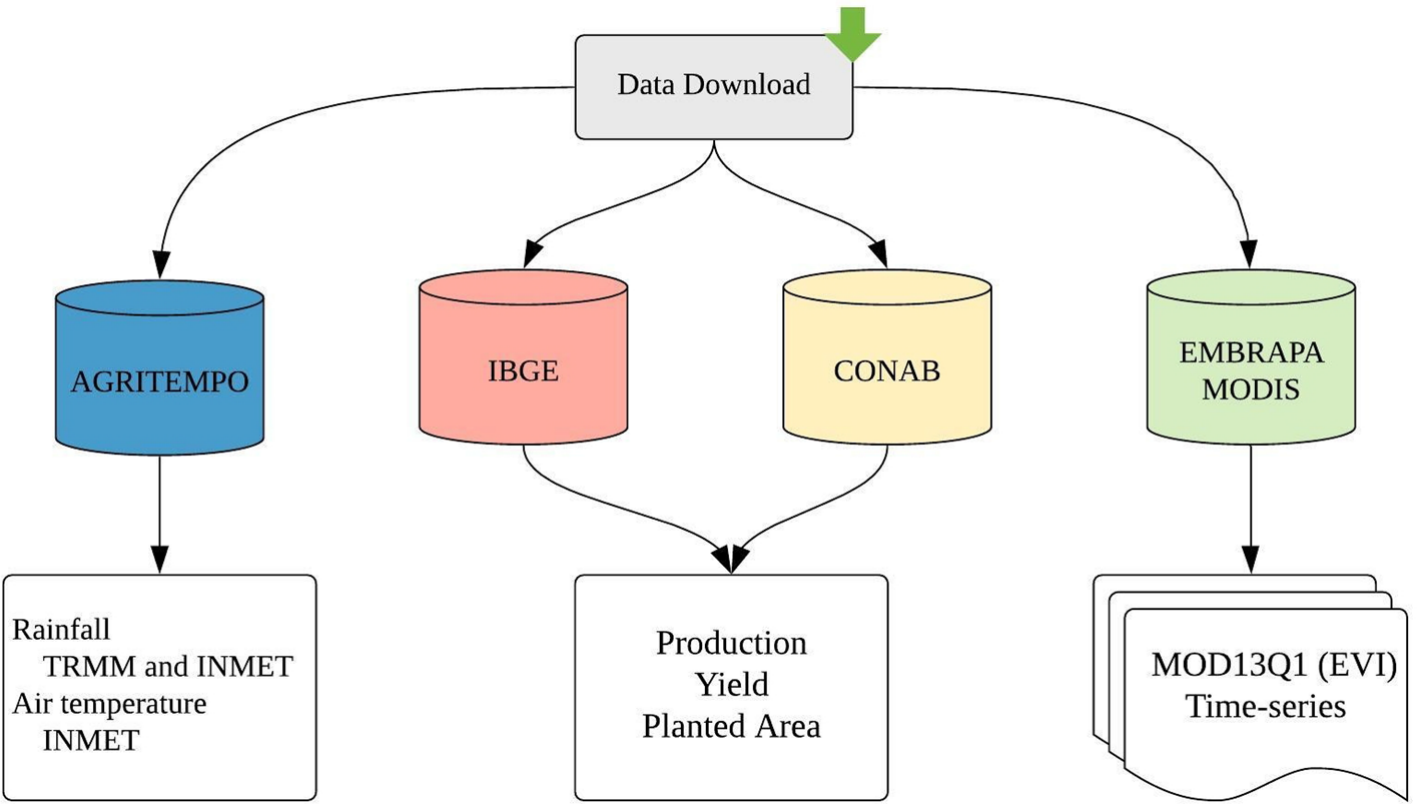
Flowchart with the source and types of data acquired.

In order to accurately correlate data of meteorological variables (rainfall and average air temperature) and surface variable (Enhanced Vegetation Index) with coffee production and yield fluctuations, data from different sources were used, namely: IBGE (Brazilian Institute of Geography and Statistics); CONAB (National Supply Company); EMBRAPA (Brazilian Agricultural Research Corporation) and AGRITEMPO (Agrometeorological Monitoring System). Figure 2 shows the flowchart with the source and the types of data acquired, which are also described in detail hereinafter.

### Data from IBGE, CONAB and AGRITEMPO

IBGE is one of the main providers of data and information in Brazil, meeting the needs of the most diverse segments of civil society, as well as the organs of the federal, state, and municipal government spheres. Data of production, yield, and planted area of the municipalities of the north, northeast and northwest of Espírito Santo (Fig. 1) for the agricultural years of 2010/2011, 2011/2012, 2012/2013, 2013/2014, 2014/2015 and 2015/2016 were used in the present study. Similarly to IBGE, CONAB conducts surveys and estimates of production, yield and planted area of the main agricultural crops in the country, but at the state and national levels. The joint use of data on a municipal (IBGE) and state/national (CONAB) scale is very important, as it enables a higher level of detail, besides being complementary. As for the data from IBGE, sequential data of production, yield and area planted of conilon coffee in the state of Espírito Santo from 2010/11 to 2015/16 were used.

Agritempo (https://www.agritempo.gov.br/agritempo/index.jsp) enables users to access meteorological and agrometeorological information from various Brazilian municipalities and states. The database of this platform comes from more than 1400 surface weather stations, conventional and automatic, stations distributed along the Brazilian territory^29^. In addition, the portal also has data from the Tropical Rainfall Measuring Mission (TRMM), a mission that was planned to estimate rainfall in tropical regions from a wide range of sensors^30–33^. The TRMM data in the Agritempo portal provides rainfall data of 11,332 grid points that are converted in the Agritempo system in the so-called virtual stations, with spatial resolution of 25 × 25 km and 30-day time resolution. On this platform, monthly data of rainfall (mm) and air temperature (°C) were acquired. Average air temperature data were only available for the municipalities of Linhares, Nova Venécia and São Mateus. Thus, in the results and discussion section, there will be only data referring to the municipalities mentioned above.

### EVI/MODIS Embrapa data

Enhanced vegetation index (EVI) images^34^ were acquired on the Embrapa MODIS website (https://www.modis.cnptia.embrapa.br). This information refers to a 16-day composition made available through the MOD13Q1 product, derived from the Moderate Resolution Imaging Spectroradiometer (MODIS) sensor on board the TERRA satellite. Embrapa MODIS aims to facilitate access to MODIS products made available by the Land Processes Distributed Active Center (LP-DAAC), providing users with ready-to-use products in state cutouts, in GeoTIFF format, in the WGS84 geographic coordinate reference system, with spatial resolution of 250 m. The MOD13Q1 product has processing level 3, which means the data are spatially resampled and temporarily composed. The temporal composition is made with pixels that contain the best possible observation over a period of 16 days based on wide observation coverage, low viewing angle, absence or shadow of clouds and aerosol^35^. A total of 144 images corresponding to the agricultural years 2010/2011, 2011/2012, 2012/2013, 2013/2014, 2014/2015, 2015/2016 were acquired.

**Figure 3:**
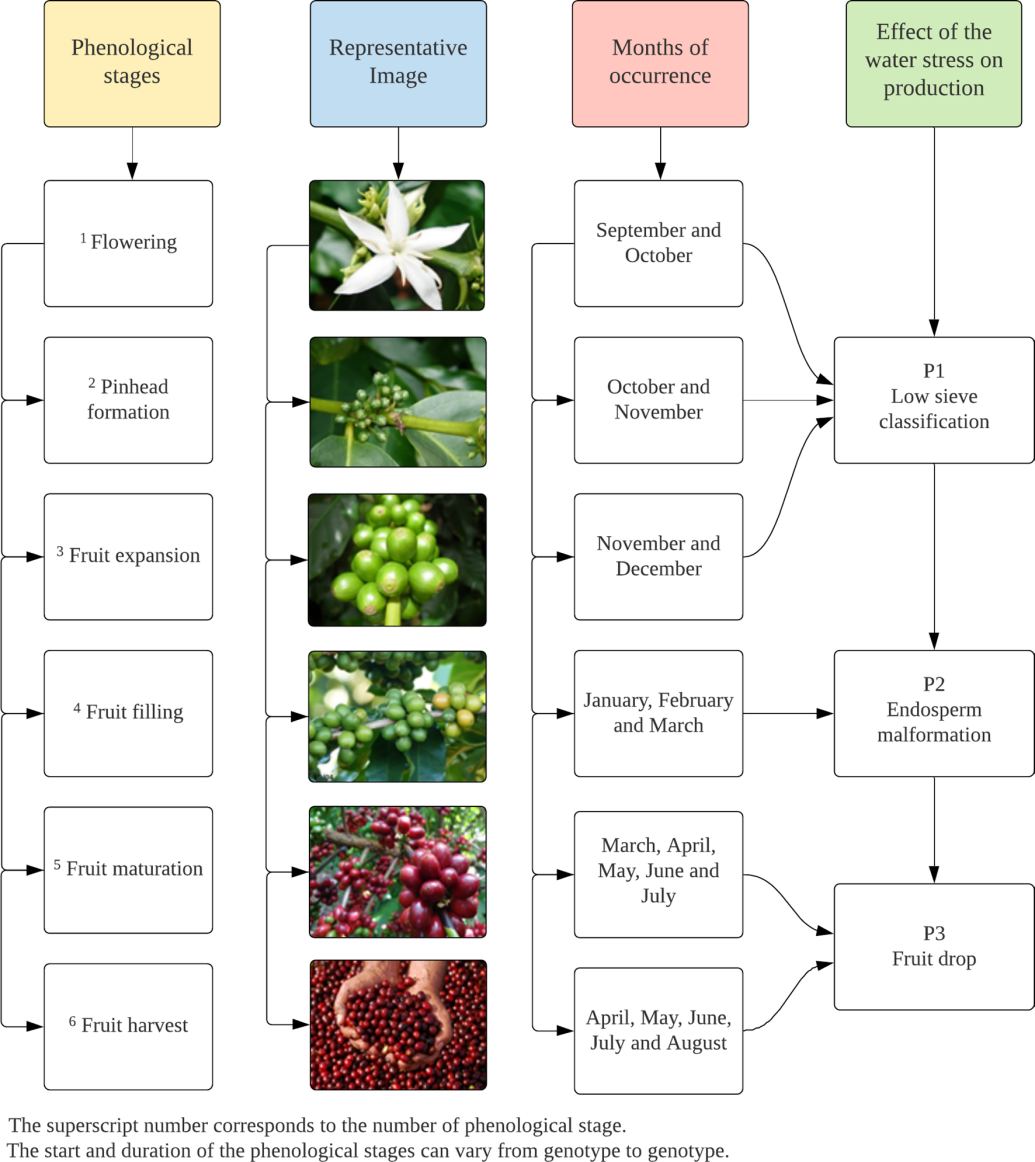
Phenological stages of the species *Coffea canephora*, months of occurrence and consequence of the water stress on production.

Each of the 144 images was rescaled to a range between 0 and 1, since they are distributed in a range of values varying from 0 to 10,000. After this, average values of EVI were obtained for the periods in which the occurrence of prolonged water deficit (WD) is critical in the production of conilon coffee for each of the six seasons. Three periods (P) were considered according to Camargo and Camargo^36^, Laviola et al.^37^ and DaMatta et al^38^: P1—September to December (WD causes the problem of low sieve classification); P2—January to March (WD causes the problem of endosperm malformation) and P3—April to August (WD causes the problem of fruit drop), as shown in Fig. 3. The information contained in this figure is extremely important to understand the effects of rainfall on coffee production and yield.

### Data analysis

First, a general description of the data of rainfall, average air temperature, yield and planted area was made for each of the six seasons analyzed in order to better characterize each season in relation to these variables. Statistically, the first analysis was the application of the model identity test^39^ to check whether the variations in production data results from either the reduction of planted area or the rainfall volume and high air temperatures. To apply the test, null (H_0_) and alternative (H_1_) hypothesis were established. H_0_ means that the parameters of the equations fitted for the annual variation of production and planted area are identical, while H_1_ means that parameters of the equations fitted for the annual variation of production and planted area are different. Thus, rejecting H_0_ means that the reduction in production is not the result of the reduction in planted area. The hypotheses were tested by analysis of variance (ANOVA), at 5% probability level (p < 0.05). The results were presented using radar charts plus the respective probability level resulting from the identity test.

Scatter plots were used to identify the impact of variations in the rainfall volume during the season (September–August) on coffee yield. The same analysis was performed for the data of average air temperature. Subsequently, the combined effects of these factors on the production were evaluated by the response surface methodology. Then, principal component analysis was carried out, which made it possible to identify among the periods (P1, P2 and P3) which is the most influential on coffee yield. Finally, maps of the mean values of EVI were generated for the study area in the periods (P1, P2 and P3) for each of the six seasons, aiming to identify the effects of drought spatially and temporally on coffee yield.

## Results and discussion

### Climatic conditions

**Figure 4:**
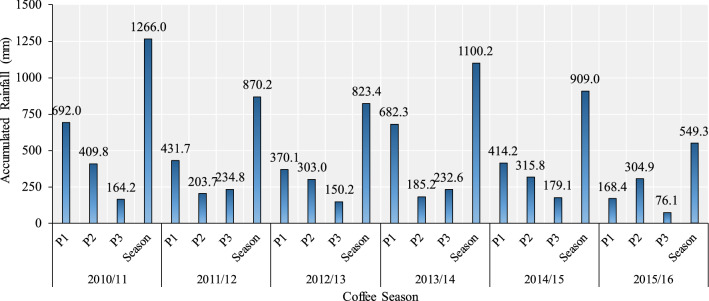
Average values of accumulated rainfall in the nine municipalities for each of the six seasons analyzed in different periods. P1: September–December; P2: January–March; P3: April–August and Season: September–August.

Figure 4 shows the average values of accumulated rainfall of the nine municipalities in different periods, for each of the six seasons analyzed. In all seasons the rainfall accumulated in the annual period (September–August) was above 823 mm, except for the 2015/2016 season, when the accumulated rainfall was only 549.3 mm. The mean of annual rainfall of six seasons was approximately 920 mm, which means that in 2015/2016 the rainfall was 40% below to mean of period, leading into a drop in production ~ 41% compared to the previous harvest. Considering that climatic conditions with rainfall of approximately 1200 mm, distributed between September and March are necessary for satisfactory development of conilon coffee^40^, the 2015/2016 season was well below what is required when rainfall is the source of water supply to plants. Moreover, the total rainfall volume in this season was much lower than historical averages, since the average minimum rainfall in the state of Espírito Santo is 1000 mm, with the highest averages (1400–1500 mm) observed in the mountainous region of the state and a downward trend in the northern region, where annual averages from 1050 to 1100 mm^41^ are observed.

It is also observed that in the 2015/2016 season the total rainfall volume (549.3 mm) was lower than the accumulated rainfall only in P1 of the 2010/2011 (692.0 mm) and 2013/2014 (682.3 mm) seasons and slightly higher than the values of the 2011/2012 (431.7 mm) and 2014/2015 (414.2 mm) seasons. In addition, during the period from April to August of the 2015/2016 season, it rained only 76.1 mm, while the average of the other seasons was 192.18 mm (Fig. 4). Regarding temporal distribution, the highest rainfalls are concentrated within P1 and P2, while in P3 the drought season prevails, a common behavior in the southeastern region of Brazil^42,43^.

The occurrence of water deficit in the April-August period (P3) favors the drop in coffee fruits, especially the smaller ones, as shown in Fig. 3, in addition to the fall of leaves. This is because the fruits, especially the larger ones, are the preferred sinks of photoassimilates during the reproductive period^37^ and, according to Lima et al.^44^, leaf fall due to water stress is a common response observed in conilon coffee clones. Although the occurrence of water deficit in the April-August period (P3) favors the drop in coffee fruits, especially the smaller ones, which is a serious problems, it is also important to highlight that a short dry period during the summer (e.g., January) is more harmful than a longer dry period during the winter. This is because in summer there is a combined effect of drought, heat, and irradiance on crop physiological performance. In the present study, these climatic variables occurred during summer in several years, which contributed to a reduction in both coffee growth and yield to a great extent.

A study of Rodríguez-López et al.^45^ with genotypes widely cultivated in state of Espírito Santo (clones 03 and 120) showed that saturating irradiance for leaf photosynthesis of conilon coffee at full sun is between 522–612 µmol photons m^−2^ s^−1^. But, under Brazilian conditions, where coffee is commonly cultivated without shading, the plants must endure irradiance as high as 2000 µmol m^−2^ s^−1^ around midday^45,46^. This means that irradiance available for conilon coffee in these locations is around 3.5 times the irradiance saturation point, a fact that further contributes to exacerbate the negative effects of drought and heat. One of the main symptoms of water deficit is a severe reduction in plant growth^11^. Plants grown under adequate water supply conditions (irrigated) are usually less resistant to water deficit and, when short water deficits occur, their morphophysiological mechanisms are severely affected because they need to quickly adapt to this deficit. Therefore, most plantations in the northern, northeast, and northwest regions of the state of Espírito Santo were severely affected, since 90% of them are irrigated[9] and did not have adequate water supply in most of the 2015/2016 season. On the other hand, it is important to highlight that this is not always the case. Overall, it is a fact that most of the productive genotypes displays a low level of drought tolerance, and therefore water stress can be harmful. However, some Conilon coffee genotypes show considerable phenotypic plasticity, as for example, a slower turgor decline with continued dehydration^12^. Therefore, a gradually imposed water deficit, as under field conditions, may allow these genotypes to have a physiological acclimatization, which can lead to short-term tolerance^10^. In this sense, in addition to the genotype’s ability, the rate of water stress imposition and duration are factors that influence how water stress affects coffee crops.

**Figure 5:**
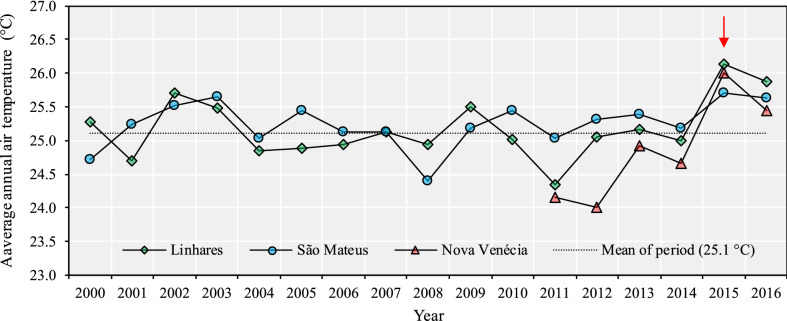
Average annual air temperature for the periods from 2000 to 2016 (Linhares and São Mateus) and from 2011 to 2016 (Nova Venécia) and the overall mean of the period.

Figure 5 shows the data of average annual air temperature for the periods from 2000 to 2016 (Linhares and São Mateus) and from 2011 to 2016 (Nova Venécia) and the overall mean of the period. In two harvest where low yields were verified (2012/2013 and 2015/2016) (Table 1), the average annual air temperatures were higher than in the previous seasons in approximately 1 °C. In addition, the highest average annual air temperature happened in the 2015/2016 season. It can be observed that from 2014, an abnormal increase in the average annual temperature (25.1 °C) began, reaching the average value of 26.1 °C in the municipality of Linhares in 2015 (Fig. 5). It is important to highlight that, even with a decrease compared to the previous year, 2016 had an average of 25.6 °C, which was higher than the average of the last 12 years. Considering the periods, the average air temperature was higher in P2 (27.3 °C), followed by P1 (25.2 °C) and P3 (23.8 °C).

**Table 1:** Yield and planted area of nine municipalities in each of the six seasons analyzed.

Municipalities	2010/2011	2011/2012	2012/2013	2013/2014	2014/2015	2015/2016
	Yield (60 kg coffee bags ha^−1^)					
Boa Esperança	39.37	39.38	36.00	36.00	29.00	15.00
Colatina	24.03	30.83	25.57	38.12	25.73	16.70
Jaguaré	35.00	39.17	25.23	39.35	29.97	17.97
Linhares	33.02	39.67	26.50	33.65	30.80	23.52
Nova Venécia	28.40	31.15	26.22	36.10	29.43	17.58
Pinheiros	41.67	40.65	29.48	36.30	28.08	24.73
Rio Bananal	30.42	38.18	35.17	37.35	25.90	20.52
São Mateus	25.00	30.80	28.00	36.00	29.00	13.00
Vila Valério	30.55	34.53	24.12	40.00	28.33	13.50
Average	31.94	36.04	28.48	36.99	28.47	18.06

Municipalities	Planted area (× 1000 ha)					
Boa Esperança	8.19	8.19	9.30	9.30	9.80	8.86
Colatina	10.50	10.50	10.53	10.47	10.47	9.86
Jaguaré	19.00	19.00	21.70	20.05	20.05	19.55
Linhares	13.82	13.80	12.50	12.50	12.50	12.03
Nova Venécia	14.30	14.30	14.10	13.80	13.80	12.50
Pinheiros	12.55	12.55	12.50	12.50	12.50	12.50
Rio Bananal	10.00	8.65	8.15	7.90	7.90	7.22
São Mateus	12.55	12.55	12.50	12.50	12.50	12.50
Vila Valério	21.50	21.50	21.10	18.80	18.80	17.10
Average	13.60	13.45	13.60	13.09	13.15	12.46

Based on the study of Eugenio et al.^47^ on agroclimatic zoning, the range considered suitable for conilon coffee cultivation varies in Brazil from 22.5 to 24 °C, so the previously mentioned temperature values are above the range considered suitable. However, other studies consider ideal the range from 22 to 26 °C^48,49^. In globally terms, conilon coffee is considered productive up to 30 °C, with optimum production between 22 and 28 °C. Regarding to minimal temperature, problems start when temperatures drop below 10 °C and the tree dies at around 4–5°C^50^. However, little is known about the effects of thermal stress on the productive performance of *C. canephora*. The studies so far have focused on identifying their effects on photosynthesis and in leaf morphological and metabolic characteristics^51^, being practically inexistent the studies that evaluated the effects of thermal stress on production. An interesting study of Kath et al.^52^ about this topic brings new insight over thermal stress on *Coffea canephora* yield. These authors used production, precipitation, and temperature data from the Southeast Asia region to model the ideal temperature range for the production of *C. canephora*, and concluded that the optimum temperature is below 20.5 °C (or an average minimum/maximum of ≤ 16.2/24.1 °C).

According to DaMatta and Ramalho^16^, drought and extreme temperatures are the main climatic limitations to coffee production. The stress caused by supra-optimal temperatures associated with water deficit in the soil and, therefore, reduction in transpiration, can intensify the occurrence of oxidative stress in coffee^16,44^. As a consequence of oxidative stress, there may be an increase in cell damage, a phenomenon known in practice as scorch^44^, which can be accompanied by leaf abscission and, if the climatic problems persist, lead to plant death. It is also worth highlighting that, besides the drastic effect resulting from the combination of drought with high temperatures, soils in the main coffee areas of Espírito Santo are acidic with low nutrient content and moderate to low water holding capacity^53^, which clearly contributed to aggravating the climatic situation.

### Yield versus planted area

Table 1 shows the values of yield and planted area for the nine municipalities in each of the six seasons analyzed. It is observed that, regardless of the municipality, the lowest values of yield among the six seasons analyzed were observed in 2015/2016, in the municipalities of São Mateus (13 bags ha^−1^) and Vila Valério (13.5 bags ha^−1^). The average yield of all municipalities in the 2015/2016 season was only 18.06 bags ha^−1^, a reduction of 36.6% compared to the 2014/2015 season. As for the planted area, there was little interannual variation, which further demonstrates the impact of drought and high temperature on coffee production.

Although the 2015/2016 season was the most affected by the climate problems, the 2014/2015 season was the first to experience the impacts of drought and high temperatures, as evidenced by the sharp reduction in yield compared to the 2013/2014 season (23.0%), even with a slight increase in planted area. Water deficit and high insolation were recorded in the 2014/2015 season, which contributed to high air temperatures from December 2014 to February 2015. This period coincides with the fruit expansion and filling (Fig. 3), which led to poor grain formation, resulting in smaller and lighter grains^54^.

In relation to the 2012/2013 season, although it received a reasonable volume of rainfall (823.4 mm) and had no problems of lack of water for irrigation, it showed low yield compared to the seasons considered as climatologically regular (2010/2011, 2011/2012 and 2013/2014). This low yield, in turn, resulted from the occurrence of intense rains at the time of flowering and fertilization of plants, thus causing a problem of fertilization and formation of fruits, impacting the final production^55^. In relation to the municipalities, the highest percentage variation in yield was observed in Vila Valério. This municipality is the second largest state producer, with 7.2% of production (Fig. 1), and showed a reduction of 52.3% in the 2015/2016 season, compared to the previous one. This municipality was one of those whose plantations had highest levels of damage due to the combined effect of water deficit and high temperature^40^.

Coffee yield in the subsequent season, as occurs with other perennial crops, is conditioned on the conditions experienced by the plantation during the previous seasons, that is, a negative impact in the previous year will have its effect on the harvest of the following year. This scenario occurred with the plantations of the north, northwest and northeast regions of Espírito Santo. From August 2014, problems of water deficit (lack of rainfall and lack of water for irrigation) and high temperatures were being intensified every month, reaching the worst scenario in the 2015/2016 season, with a sharp reduction of yield (Table1). In this season, the state production was only 5 million and 35 thousand 60 kg coffee bags, the lowest harvest since 2004^26^.

### Annual variation of production versus annual variation of planted area

**Figure 6:**
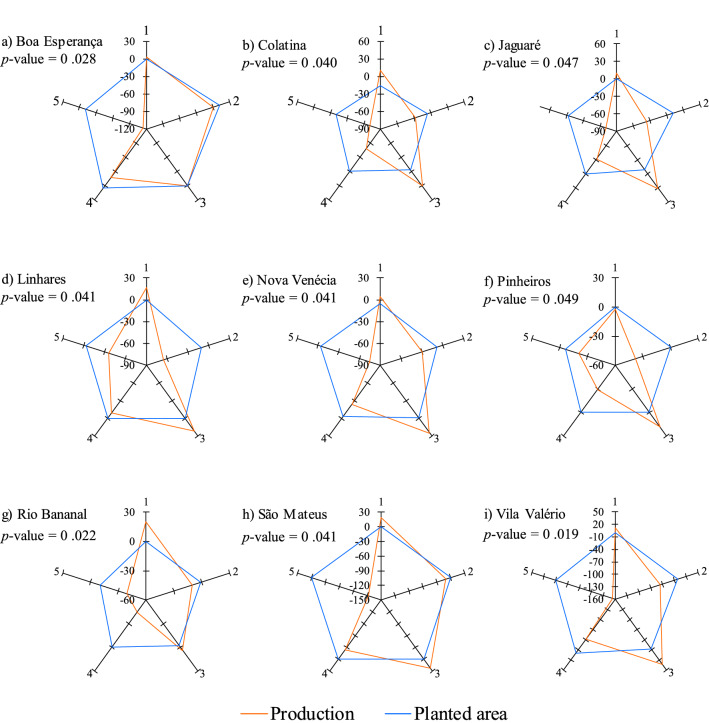
Radar charts with the respective probability levels resulting from the identity test of the regression model for the variables: (i) annual variation of production and (ii) annual variation of planted area of nine conilon coffee-producing municipalities in the state of Espírito Santo. Seasons compared: (1) 2011/2012 with 2010/2011; (2) 2012/2013 with 2011/2012; (3) 2013/2014 with 2012/2013; (4) 2014/2015 with 2013/2014 and (5) 2015/2016 with 2014/2015.

Figure 6 shows the radar charts along with the respective probability levels resulting from the identity tests of the regression model for the variables: (1) annual variation of production and (2) annual variation of planted area. It is verified that, for all nine municipalities analyzed, the models were different (p-value always lower than the significance level of 5%), which makes it possible to affirm that the reduction of production does not result from the reduction of planted area. It is also possible to confirm the statistical results visually, since a drastic reduction in production values is clearly observed from the compared seasons 4 and especially 5, while the area in the same period remained virtually without variations (Fig. 6). From this finding, the focus becomes the identification of the effects of the accumulated rainfall volume and average air temperature at different stages of the phenological cycle of conilon coffee and, consequently, the impact on its production and yield.

The present study disregarded the effect of biennial bearing, defined as annual alternation of high and low coffee yields, on production^56^. This is because in conilon coffee biennial bearing is minimized or buffered, within certain limits, when compared to Arabica coffee, due to the periodic renewal of orthotropic stems, through an intense and well-planned pruning system^57^, and also due to other management practices, including irrigation^58^. In addition, the quantification of biennial bearing in *Coffea canephora* plantation is still very complex because of two main reasons. The first is due to the characteristic of cross fertilization of the plants, which requires the planting of several genotypes in the same plot for effective fertilization, thus leading to a great mixture, which makes it very difficult to determine biennial bearing. The second point is that biennial bearing occurs among plots, among plants and within the same plant.

### Effect of annual accumulated rainfall on yield

**Figure 7:**
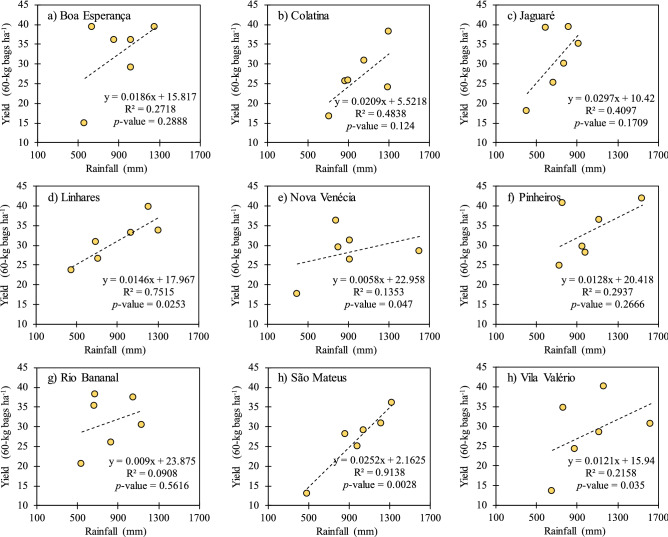
Relationship between the rainfall accumulated during the season (September–August) and coffee yield.

Overall, agricultural production is directly proportional to the rainfall volume, where this relationship is more evident in non-irrigated crops. In this sense, the low and insignificant relationships between precipitation and production in this study (Fig. 7) demonstrate the effect of irrigation on the performance of other environmental factors. Figure 7 shows the relationship between accumulated rainfall by agricultural year (September–August) and coffee yield. Although there is statistical significance only for the regressions of the municipalities of Linhares, Nova Venécia, São Mateus and Vila Valério, there is clearly a tendency of increase in coffee yield with the increase in rainfall volume in all nine locations. The trend becomes a very important point besides the significance of regression when working with large-scale data (e.g., municipal production), since on this scale there are many factors that can interfere in yield besides rainfall. Craparo et al.^59^, for instance, evaluated the effect of several rainfall variables on the production and yield of Arabica coffee in several districts of Tanzania in Africa and found that the only rainfall variable that is slightly correlated with production and yield is the number of days of rainfall in the flowering period, although it is not statistically significant.

According to DaMatta et al.^38^, intense water deficit in conilon coffee leads to accelerated dehydration of tissues, causing collapse of metabolism, culminating in considerable loss of leaf area and, if the problem persists, production is irreversibly affected. Therefore, the authors point out that conilon coffee clones cultivated in Espírito Santo, in general, require the implementation of irrigation for economic exploitation. An important point in this scenario of production/yield reduction refers to the availability of water for irrigation. The study region has as one of its main characteristics the adoption of irrigation to meet the water requirement of plants. However, rainfall volumes below historical averages between August 2014 and September 2016 resulted in the depletion of the main sources of water for irrigation^40^, that is, water available in small and medium-sized rivers, streams and small earth dams.

Unlike irrigation, which enables water application in the correct quantity and at the correct time for a given crop, rainfall is a climatic variable with high spatial–temporal variability. This characteristic occurs due to a combination of factors, including climatic conditions, rainfall generation mechanisms, topographic characteristics, land use and proximity to the sea and other water surfaces^60,61^. Understanding this variability of rainfall has always been a major challenge, and the impacts of climate change further complicate this issue^62^. Therefore, the discussion about the effect of this variable on yield should be cautious in order to avoid false conclusions.

### Effect of average annual air temperature on yield

**Figure 8:**
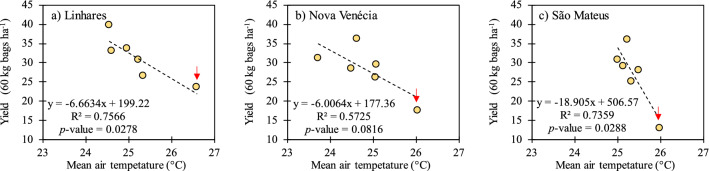
Correlation between the mean air temperature and conilon coffee yield in the municipalities of (**a**) Linhares, (**b**) Nova Venécia and (**c**) São Mateus.

The air temperature increases above 25 °C resulted in a decrease in production (Fig. 8). The relationships were more evident and significant than rainfall, since the increase in air temperature can affect crop yields regardless of the water availability in the soil^63^. However, the effects may be exacerbated under conditions of drought and high irradiance. Figure 8 shows the impact of the increase in average air temperature in each season on conilon coffee yield. As for the data of accumulated rainfall, it is possible to verify the sensitivity of coffee yield to temperature, in the present case the increase of this variable leading to a decline in yield. The red arrow refers to the average temperature of the 2015/2016 season, equal to 26.6 °C in the municipality of Linhares (Fig. 8a) and to 26 °C for the municipalities of Nova Venécia and São Mateus (Fig. 8b,c).

For the three municipalities the average annual temperature in 2015/2016 was higher than ideal range, considering values used by Eugenio et al.^47^ in their study about zoning agroclimatological *Coffea canephora* for Espírito Santo (22.5–24 °C) or very close to the superior limit, considering data of Matiello^48^ and Taques and Dadalto^49^, that’s of 22–26 °C. To increase the problematic, the 2015/2016 season was accompanied by droughts, leading to a sharp reduction of yield in the 2015/2016 season (Table 1). In the modeling study by Kath et al.^52^, the authors concluded that an ideal temperature range above 22 °C is probably overestimated. On the other hand, we believe that the conclusions of these authors are specific to the region where the data were collected and cannot be directly applied to the species cultivated in other regions of the world, especially in Brazil.

Outside their optimum temperature ranges, the bean quality of both species declines, as does yield. Changing climate might also increase exposure and vulnerability of coffee to pests and diseases^64^. However, it's important to point out, that in the last two decades occurred great genetic advancement in conilon coffee growing, producing genotypes with different characteristics, mainly in terms of yield^65^. Therefore, the ideal temperature range for a genotype, may not be the same for another, although the trend be very similar intervals.

Gay et al.^66^*,* working with modeling of *C. arabica* production as a function of climate changes, concluded that air temperature is the most relevant climate factor for its production, since it responds significantly to the seasonal patterns of temperature. On the other hand, there are practices, such as agroforestry shading, that are very efficient to avoid this problem of high temperatures, since they act in reducing air temperature and direct solar radiation on the canopy of plants, without compromising yield, as observed in some studies with conilon coffee^67–70^.

Recent decades have been characterized by increasing temperatures worldwide, which also resulted in an exponential increase of VPD^71,72^ that can be very harmful to coffee, since it is a crop very sensitive to this climatic variable^73^. Barros indicated VPD values of 2.2 kPa as the upper limit for coffee plants, as values above that limit can result in decrease of stomatal and canopy conductance in *Coffea* spp. More recent evidences show that when VPD exceeded 2 kPa, there was a considerable decrease in canopy conductance^74^. The Coffee spp. sensitivity to high VPD values offer crucial evidence that climate changes can be harmful to coffee plants, mainly for C. canephora genotypes, which are drought-sensitive.

On the other hand, drought-resistant genotypes can be benefited because they generally show reduction of yield under optimal environments conditions. This happens for such genotypes due to the increase of sensitivity of their stomata to VPD^75^. Thereby, these authors concluded that coffee genotypes displaying increased phenotypic plasticity (e.g., deep root system, substantial hydraulic conductance, intermediate stomatal control and strengthening of antioxidant defense system) could be used in regions which are predicted to face moderate water deficit, while drought-resistant genotypes could be used in regions predicted to face severe drought. In addition, Rodrigues et al.^76^ suggested that the coffee genotype, for global warming conditions, must have the ability to transport water throughout the plant system, maintaining adequate leaf water content and maximizing stomatal opening.

### Combined effect of accumulated rainfall and air average temperature on coffee production

**Figure 9:**
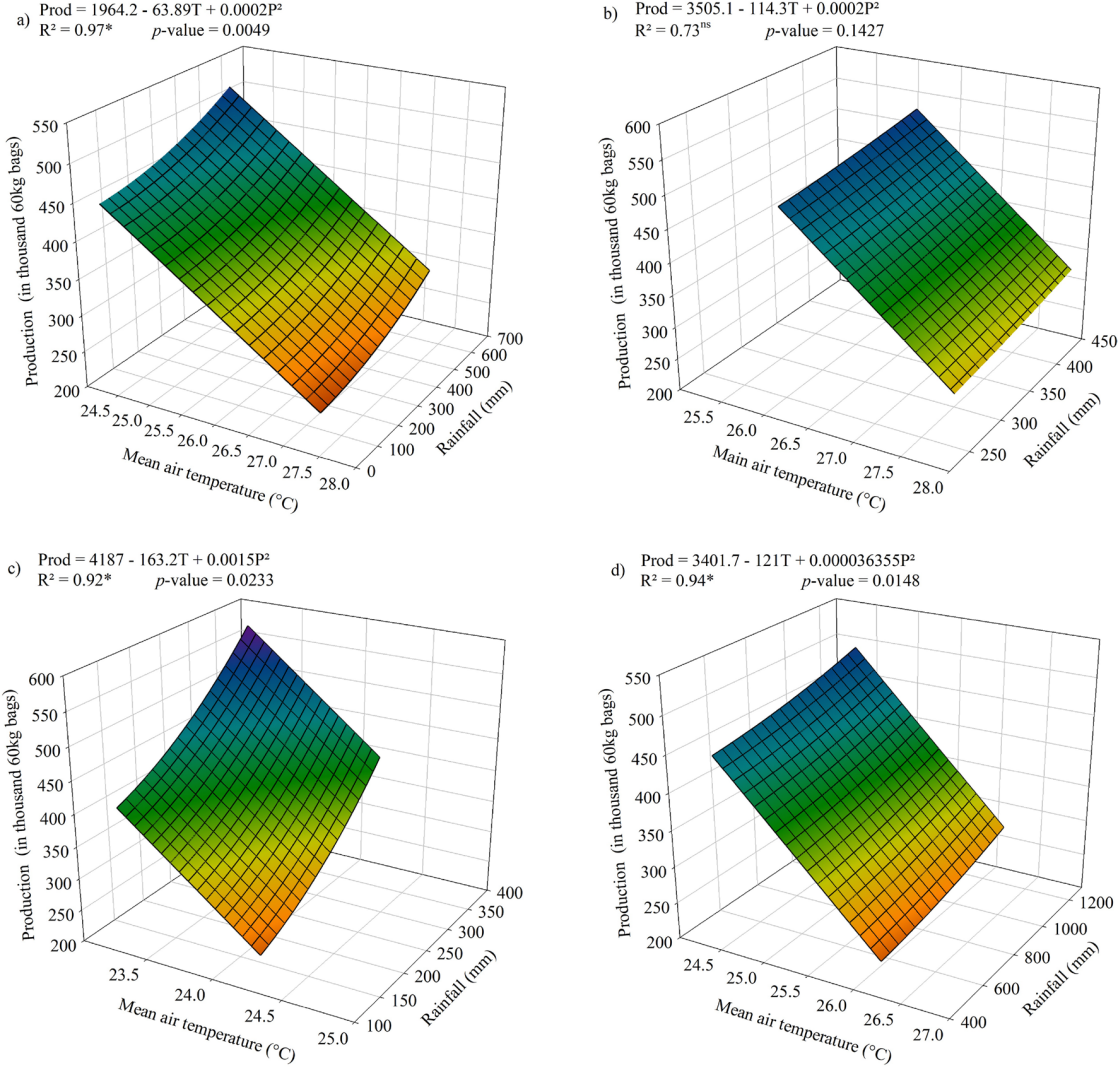
Production of conilon coffee in response to the accumulated values of rainfall and mean air temperature for the periods of (**a**) September–December, January–March (**b**), April–August (**c**) and annual period (**d**). *significant at 5% probability.

Figure 9 shows the graphs of response surface resulting from the combined effect of accumulated rainfall and temperature on coffee production. Before delving deeper in the topic discussion, it is important to highlight that the variations in planted area were small and rather insignificant (Fig. 6), favoring the study of the impacts from variations in temperature and precipitation on crop production. There was a significant interaction between the average air temperature and the accumulated rainfall for coffee production in the periods of September–December (Fig. 9a), April–August (Fig. 9c) and annual (Fig. 9d), and the reduction of production according to the increase in temperature and reduction of accumulated rainfall, as expected. Thus, greater decreases in production can be seen when increases in temperature are associated with lower rainfall.

There was great similarity between the response surfaces for the Period January–March (Fig. 9b) and annual period (Fig. 9d), with a virtually linear reduction in production. It can be observed that accumulated annual rainfall close to 700 mm and air temperature above 25.5 °C caused annual production lower than 250 thousand 60 kg coffee bags (Fig. 9d). The January–March period was not significant (Fig. 9b), but an analysis of it is quite valid, because this is the period where conilon coffee is found in the fruit filling stage and, if water deficit occurs, the problem of endosperm malformation may occur (Fig. 3).The coffee producers have a clear understanding of the importance of adequate water availability during this phenological phase (grain filling phase). Because of that, they focus on an efficient irrigation management during this stage, which helps to explain the lack of significance. In addition, it should be noted that, although the model is not significant, which is largely due to the small effect of precipitation as previously explained, there seems to be a sharp decrease in production with increasing temperature, regardless of precipitation.

Considering the annual regression model (Fig. 9d) and the 1 °C increase in the average annual air temperature, the production decreases: (1) 31% considering the average annual precipitation of 920 mm; (2) 25% considering a rainfall of 1200 mm; (3) 36% considering the 50% decrease in average annual precipitation. In the modeling study performed by Kath et al.^52^, the increase of 1 °C in the average air temperature (minimum and maximum) caused a decrease in production of approximately 14%. Thus, increases in air temperature have a higher impact (negative) on production in comparison to decreases in annual precipitation. It is noteworthy that in the northern, northeastern and northwestern regions of Espírito Santo, approximately 90% of crops are grown using irrigation^9^ in addition to the use of varieties that have some degree of drought tolerance^77,78^, the main target for improvement in recent decades.

In Brazil, heat tolerance has not yet been considered in the genetic breeding studies of *C. canephora*. These results demonstrate, in a broad way, the sensitivity of the conilon coffee plantations to the increase of the air temperature. Differently to the evidence raised by DaMatta et al.^79^, the effect of the temperature increase seems to negatively affect production, even under conditions of adequate water supply. Furthermore, considering the likely impacts of climate change, we believe that in *C. canephora* plantations, the potential positive effects of increasing the concentration of atmospheric CO_2_ to mitigate the negative impact of rising temperatures^80,81^ will not be effective. As proposed by Rahn et al.^82^, the effects of CO_2_ concentration will be more significant, especially at higher altitudes and, therefore, more evident in *C. arabica* specie.

High temperatures in the period from September to December, which encompasses the flowering and early fruiting stages (Fig. 3), may lead to the abortion of flowers^36^, while water deficit causes the problem of low sieve classification (smaller fruits), hence causing a strong impact on production. In addition, high temperatures may lead to an early maturation of the fruits due to the earlier break of dormancy of the buds^83^. High temperatures in the period from January to March could lead to a quality problem due to early and excessive ripening of fruits^83^. These authors point out that coffee is very resistant to the high temperature of summer and drought, but the increase in extreme conditions may be responsible for physiological stresses, such as reduction in photosynthetic efficiency. These significant relationships reinforce what has been demonstrated by some studies^59,66^, that increased air temperature has a significant influence on the physiology of growth and production in coffee plants at each of the phenological stages.

Bunn et al.^84^ conducted a study on the profile of climate changes in the global production of arabica and conilon coffee and highlighted that coffee has proven to be highly sensitive to climate change, in addition to the fact that dominant production regions in the world (Brazil and Vietnam) may experience substantial reductions in the areas available for coffee. Additionally, Pham et al.^64^ highlights that as a climate-sensitive perennial crop, coffee is likely to be highly susceptible to changes in climate.

### Principal component analysis: identification of the most influential period of rainfall accumulation on yield

**Figure 10:**
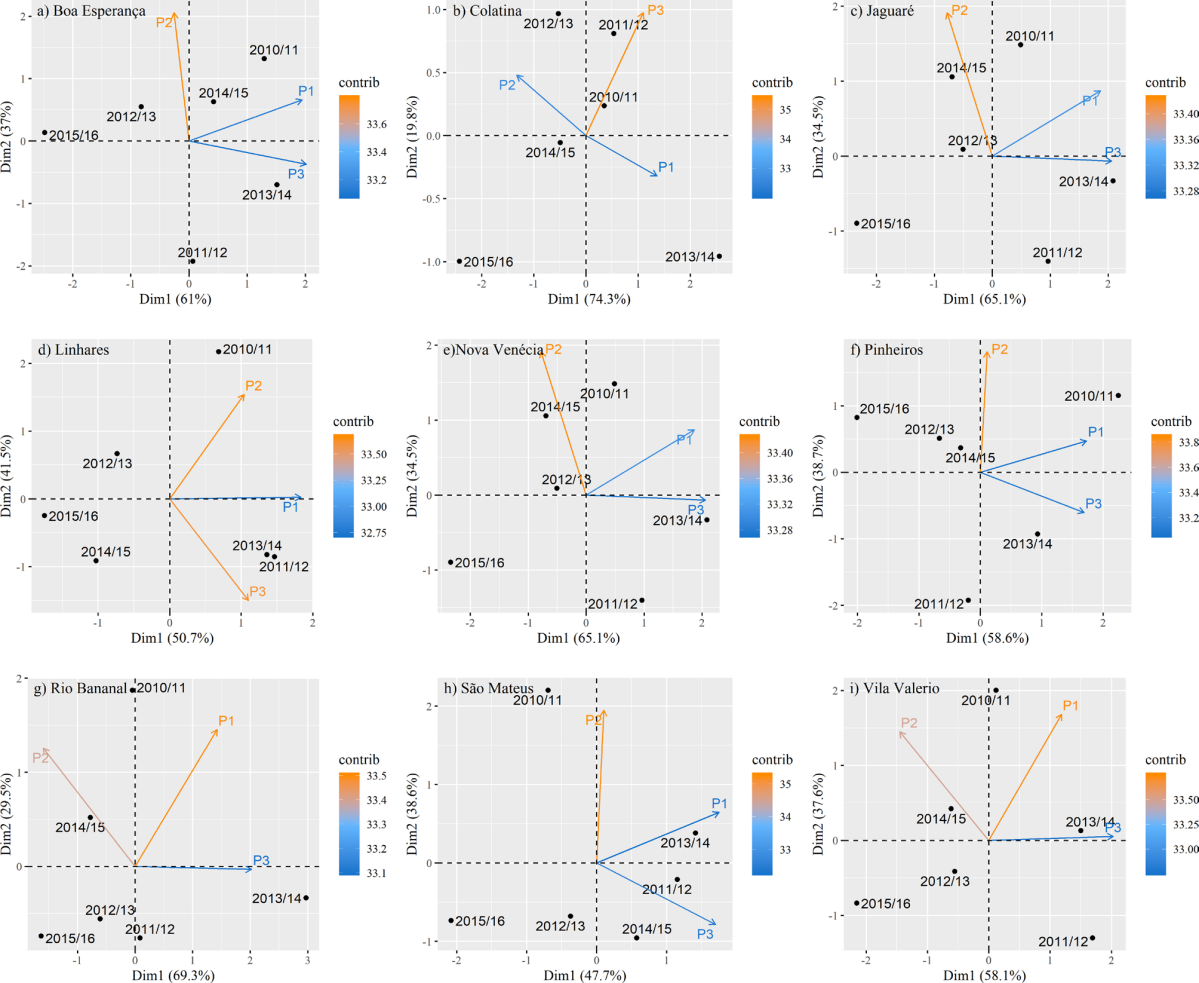
Biplot with values of accumulated rainfall (variables) for the periods September–December (P1), January–March (P2) and April–August (P3) and with six seasons (individuals) for each of the nine coffee-producing municipalities.

Figure 10 presents a biplot of the relationship between the three periods of rainfall accumulation (variables) and the six seasons (individuals) for each of the nine municipalities, where the principal component 1 (Dim1) is represented by the X-axis and principal component 2 (Dim2) by the Y-axis. A general analysis involving all nine municipalities, based on the components, showed that the highest coffee yields are correlated with the rains that occurred in the periods P1 and P3, since these appear predominantly in the first and fourth quadrants, which are the most associated with the first component, concentrating the three major seasons, i.e. 2013/2014, 2011/2012 and 2010/2011, among the six studied. P2 was present in the second quadrant six times (Fig. 10a–c,e,g,i), so January–March is a period less associated with Dim1; therefore, it can be said that this period is the one that has the least influence on coffee yield.

Regarding the results of the principal component analysis, it is important to highlight that, in order to draw a better conclusion on the effects of accumulated rainfall or water supply on coffee yield, it is necessary to conduct an experimental study preferably in a controlled environment, where the applied water depth can be controlled. In this line, there are several studies been performed with the conilon coffee and, the results have been show that water deficit causes on plants suppression of net photosynthesis^44^ or the progressive reduction of this^85^, decreases in stomatal conductance^11^ and, decreased carbon assimilation^86^. However, it’s believed that there exists a wide range of genetic variability among conilon coffee clones for traits associated with drought tolerance^85^. In addition, as the varieties of conilon coffee comprise several genotypes^87^, with high genetic variability^88–90^, they may show different responses for the same treatment.

### Detection of the drought and heat effects over coffee plantations using Enhanced Vegetation Index (EVI)

Drought is one of the most destructive natural disasters, and numerous studies predict it will become more severe and widespread under climate change^91^. Thus, acquiring knowledge on the impact of this phenomenon is essential for several decision-making processes. In large areas (like in present study), remote sensing-based vegetation indices (VI) are a key tool for this purpose. VI that make uses red and near infrared wavelengths in their calculations, such as the EVI, are very sensitivity to plant leaf area^92–94^ and photosynthetic pigments^95–97^ variations, which in turn, can be strongly affected by drought, heat, and excessive irradiance. In this sense, using the EVI conilon plantations is particularly useful to reflect some possible physiological disorders caused by drought and heat, for instance, fall of leaves, pigment degradation, and limited nutrient absorption capacity.

**Figure 11:**
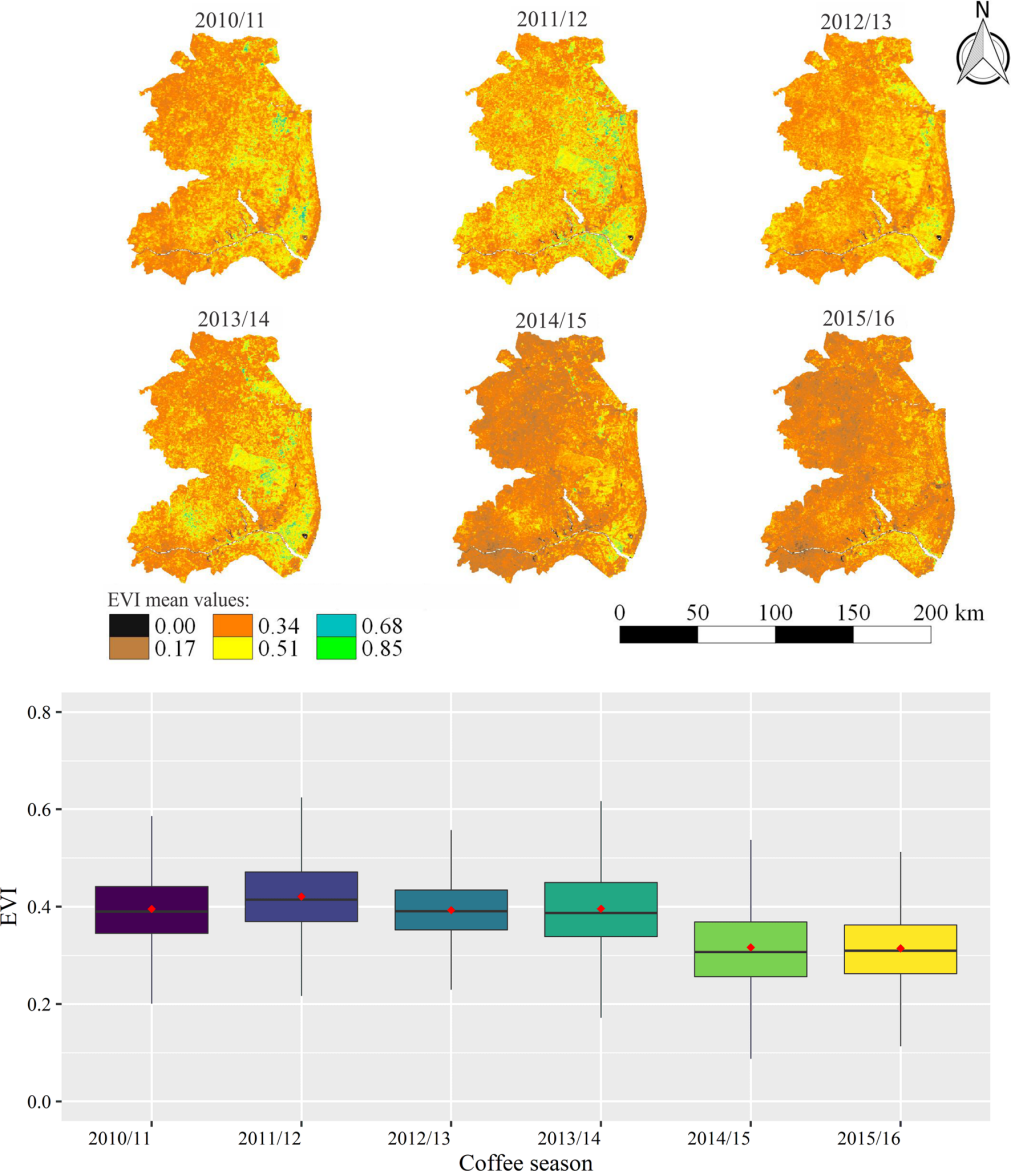
Average values of EVI for the northern, northwest and northeast regions of the Espírito Santo state, in the period from September to December (P1) of six different seasons, in which the occurrence of water stress causes the problem of low sieve classification in conilon coffee.

Figure 11 presents information on the variations in EVI values along the six seasons analyzed for the period from September to December (P1). Based on visual interpretation (maps), lower EVI values is an evident similarity between the 2014/2015 and 2015/2016 seasons. Through the boxplot, it is possible to observe that the impact of drought and heat is more pronounced in the north, northwest, and northeast regions of Espírito Santo. The mean values of EVI (red points) were close to 0.35, while, for the other seasons, they remained near to 0.40. The stress caused by supra-optimal temperatures can cause photosynthetic pigment degradation^76^ and, when associated with water deficit in the soil, it can lead to a reduction in transpiration which can intensify the occurrence of oxidative stress in coffee^16,44^. As a consequence of oxidative stress, there may be an increase in cell damage, which can result in leaf abscission and even plant death (in severe cases)^44^. Such outcome will directly reflect in EVI values, given their large sensitivity to leaf area index and photosynthetic pigments.

**Figure 12:**
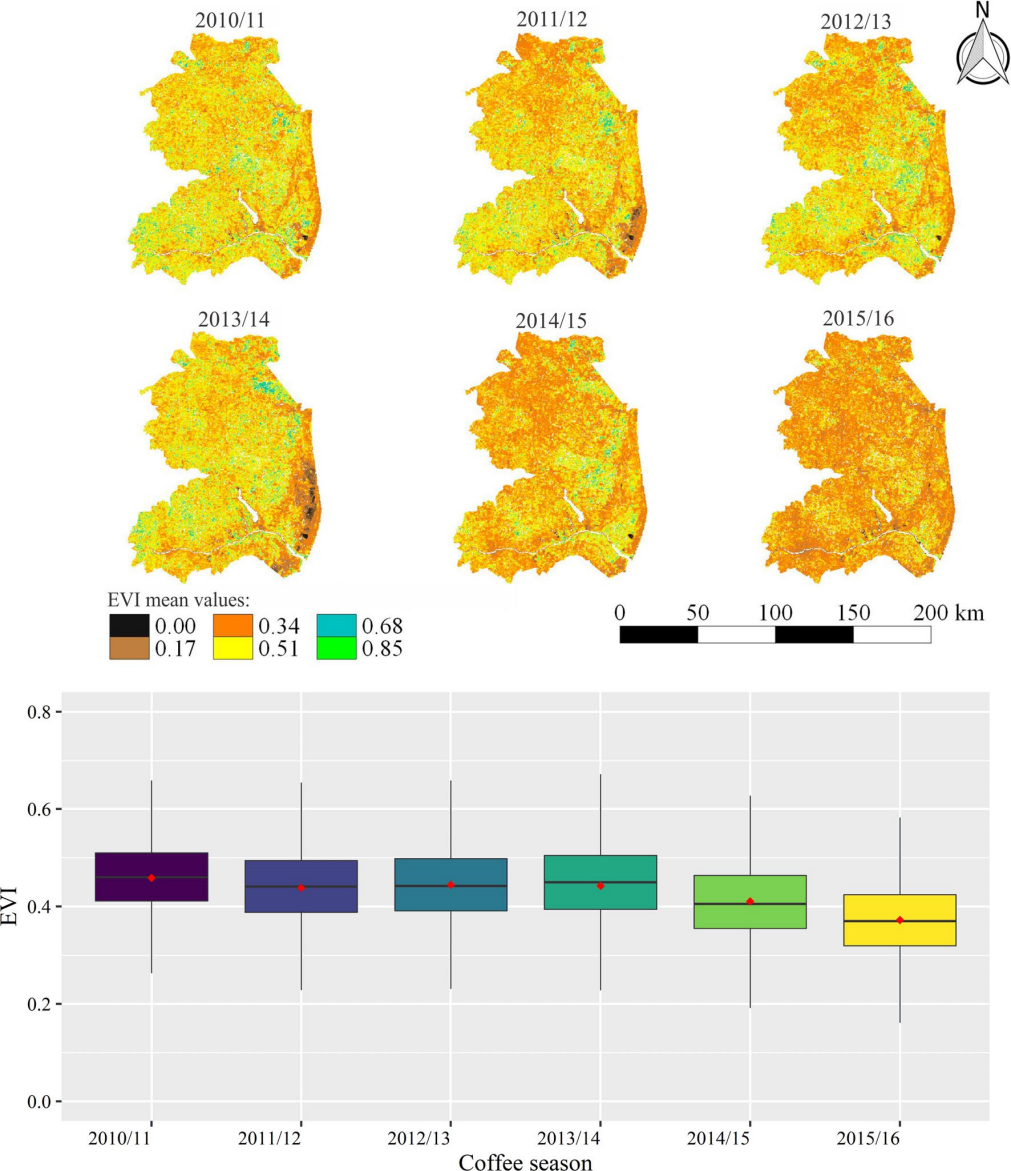
Average values of EVI for the northern, northwest and northeast regions of the Espírito Santo state, in the period from January to March (P2) in six different seasons, in which the occurrence of water stress causes the problem of endosperm malformation in conilon coffee.

Figueira Branco et al.^98^ used the EVI derived from the MODIS sensor to detect the occurrence of droughts in areas of tropical forest, which also represents the present study area, and concluded that EVI data are able to show the response of the vegetation under drought conditions. Several similar studies have been conducted using EVI for drought detection and monitoring^91,99–102^. As evidenced in Fig. 11, in P1, the lowest values of EVI are also observed for the 2014/2015 and 2015/2016 seasons in P2, from January to March (Fig. 12), but with greater discrepancy between these seasons than in P1. By comparing the periods (P1 and P2) for the same season (Figs. 11 and 12), it is possible to observe that the mean values of EVI in the January–March interval (P2) are higher than those in September–December (P1). This fact occurs because soon after harvest (April–August, Fig. 3), plants have a lower number of leaves due to the loss during fruit harvesting and rejuvenation pruning, resulting in lower leaf area index and, consequently, lower EVI values. Also, according to these authors, plants cannot recover the leaves lost through new shoots in a sufficiently short time to ensure good flowering, due to the established stress. Thus, the use of irrigation is essential in this period of the year to provide water for plants so that they can recover.

**Figure 13:**
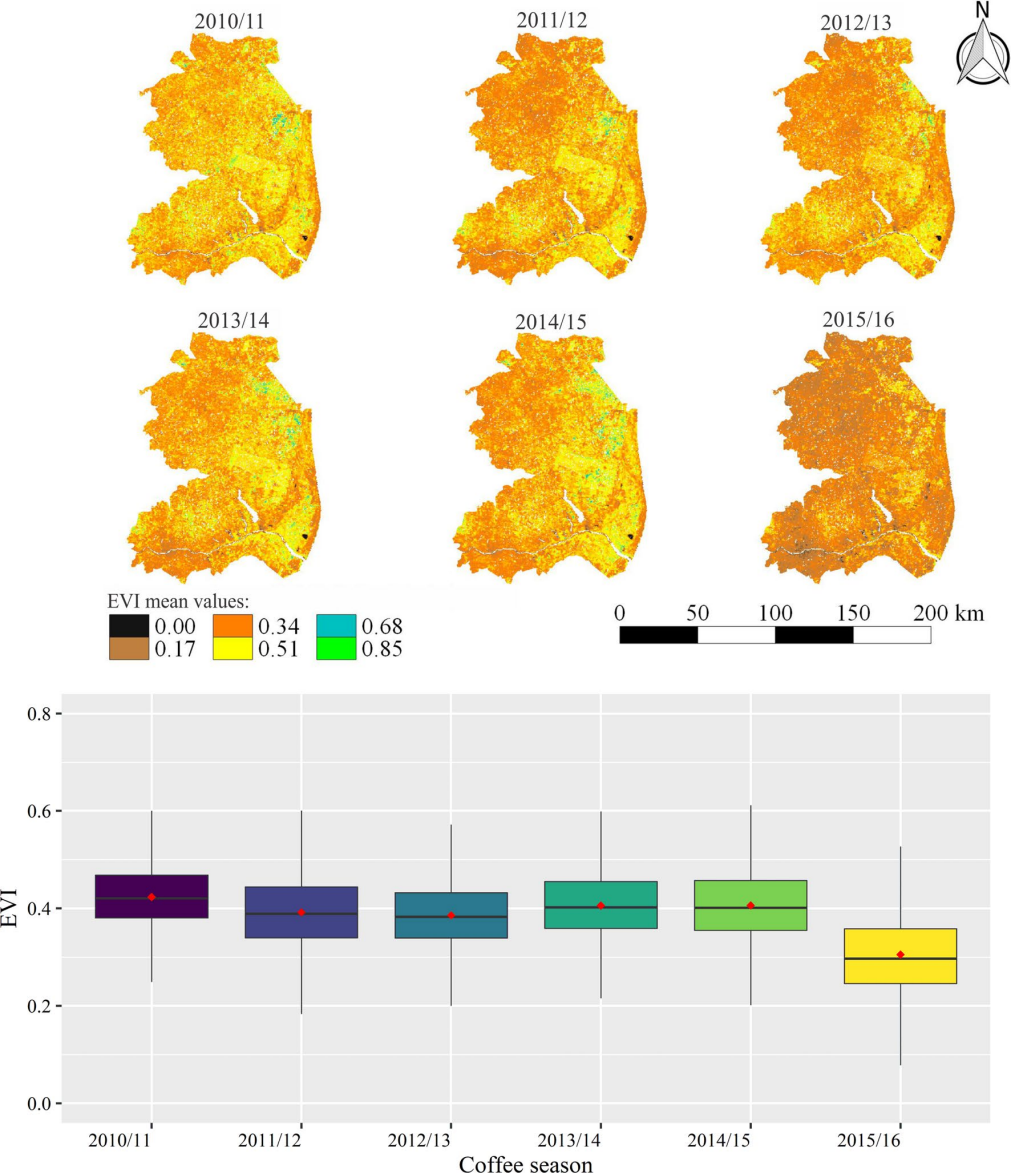
Average values of EVI for the norther, northwest and northeast regions of the Espírito Santo state, in the period from April to August (P3) of six different seasons, in which the occurrence of water stress causes the problem of fruit drop in conilon coffee.

In the period from April to August, as well as for the other periods, the greatest impacts of drought were observed in the 2015/2016 season (Fig. 13). According to the boxplot, this impact more pronounced where the average values were around 0.35 for this season. For the 2014/2015 season, the behavior of P3 with EVI values close to 0.4 differed from the pattern found in the periods P1 and P2, which had average values around 0.35. This can be explained by the fact that the rains during the 2014/2015 season exceeded 900 mm and irrigations were still carried out with some frequency.

Data available on the website of Capixaba Institute of Research, Technical Assistance and Rural Extension (INCAPER) also help to highlight the drought that occurred in the north, northwest and northeast regions of Espírito Santo. This institute has an agrometeorology sector, which provides spatialized data of water balance and the Standardized Precipitation Index (SPI)^103^ or the entire state on a monthly scale. The Incaper data of SPI for 2015 showed that virtually the entire area of the present study was classified as extremely dry^104^. Regarding the water balance in the rainy season (October–February), also for the year 2015, the values were negative or positive close to zero, with a slight increase during the season considered dry (March–September)^105^.

Climate problems have dramatically affected the production and consequently the price of coffee. According to data from the Brazilian Association of the Coffee Industry^106^, in 2016 the average price of the 60 kg coffee bag (conilon type 7) marketed in Espírito Santo was R$ 410.45, whereas in 2015 the value was R$ 315.15. This price increase was extremely important for coffee growers in the region, as most of them were decapitalized due to the situation faced. A worrying scenario for the state of Espírito Santo is that conilon coffee is the main source of income in 80% of rural properties located in the north, northwest and northeast regions of the state^9^. Thus, problems that strongly impact coffee production will lead to social and economic losses for families in these regions. From a socio-economic perspective, understanding the extent of climate-driven impacts on coffee production and the benefits of potential adaptation strategies will be of vital importance to maintaining and improving coffee productivity and profitability and sustaining the livelihoods of smallholder producers all over the world^64^.

A recent study of Magrach and Ghazoul^7^ shows that conilon variety could lose 55% of currently suitable areas (mostly within western Africa and Brazil), but the future suitable area is expected to more than double, particularly by the extension of climatically suitable conditions in the Amazon Basin and South East Asia, which together will total 97.4 million hectares. The authors also mention that climate change will be detrimental for Arabica cultivation, though the area suitable for Robusta will increase greatly by 2050. Both Arabica and conilon will be subject to important geographic shifts in their distribution. Thus, several coffee producers of Espírito Santo state can be affected.

Because of this, it is important that government encourage coffee growers to diversify their production so as not to obtain their income from a single source, and also encourage them through technical training that helps cope with climate problems, such as agroforestry shading. Moreira et al.^19^ compared the traditional planting of coffee without shade and shaded with macaúba palm (*Acrocomia aculeata*) and observed in the intercropped planting higher soil moisture content at depths from 20 to 40 cm, higher photosynthetically active radiation and maximum air temperatures below 30 °C. These authors concluded that agroforestry system with coffee and macaúba palm can be a strategy of adaptation under future climatic variability and changes related to high temperatures and low rainfall. According to^107^, agroforestry systems offer a promising strategy for coffee growing in the Atlantic Forest biome region, which comprises the state of Espírito Santo. However, in Brazil predominates a paradigm of that the shading in coffee conilon is synonymous of less yield^108^, what hinders the adopt an agroforestry system by farmers, which is not true. According to Venancio et al.^108^, the success of shaded plantations depends the choice of the shade responsive genotypes and appropriate shading level for it.

Another alternative would be to build small reservoirs, which can be an excellent option to increase water supply during drought periods^109^. According to Ogilvie et al.^110^, in semiarid regions, small reservoirs have the potential to support the techniques of subsistence agriculture of small farmers. Overall, this study shows that drought and high temperatures impacted the species *Coffea canephora* between August 2014 and August 2016 in the northern, northwest, and northeastern regions of the state of Espírito Santo. Our study also demonstrated that a combination of climatic data (rainfall and air temperature) and vegetation indices (e.g., EVI) with crop data (production, yield and planted area) provide information that, when correlated, is able to enhance the understanding of phenomena such as drought and its consequences on crops.

Finally, drought is a global issue that has gained increasing attention from many countries and international organizations and should not be seen simply as a merely physical phenomenon^111^, but rather as one a repetitious phenomenon can affect a large area over a period of time, which can last from a few months to several years^112^ and, and, that have always been part of human history, and when combined with social or political failures they have been linked to civil unrest, famines and even the collapse of civilizations^113^. Thus, it is essential that all drought-prone nations share lessons learned in drought risk management if it wants to make progress in reducing the vulnerability to this problem^111^. The drought monitoring also is essential to helps decision makers to achieve a better insight into conditions^112^. Besides that, there are a decreasing bioclimatic suitability for Robusta production projected in some global studies, further research for this coffee species, particularly at finer spatial scales is necessary^64^.

## Conclusions

Our study explored the combination of various data (rainfall, air temperature, production, yield, planted area and surface reflectance data) from various sources to understand the real impact of drought in the main conilon coffee-producing region of the state of Espírito Santo and also Brazil. The 2015/2016 season was the most affected by droughts and high temperatures because, besides suffering from the impacts of adverse weather conditions throughout its duration, coffee plants were already weakened by the climatic conditions of the previous season (2014/2015). Regardless of the rainy period (P1, P2, P3 or the entire season), the increase of temperature with reduction of rainfall causes a reduction in coffee production.

The rains that occurred in the September–December (P1) and April–August (P3) periods had greater influence on coffee yield according to the principal component analysis. However, it is important to highlight that a short dry period from January to March (P2) is more harmful than a longer dry period during the winter (P3). P2 occurs in the summer season, where generally there is a combined effect of drought, heat, and high irradiance on crop physiological performance. However, specific research is necessary for this conclusion, particularly at controlled environment. We verified that the increase in air temperature has an higher negative impact on crop production than the decrease in annual precipitation. In addition, in the two harvest seasons that stood out for the lowest yields (2012/2013 and 2015/2016), the average annual air temperatures were higher in approximately 1 °C than in the previous seasons. In the 2015/2016 season, the average annual air temperature reached the highest value of the entire series.

This study also highlights the importance of integrating spatialized information from a vegetation spectral index (EVI) with rainfall and air temperature data to evaluate coffee yield variations under conditions of drought and high temperatures. The conilon coffee plantations of the north, northwest and northeast region of the state of Espírito Santo are susceptible to new climate extremes, as they continue to be managed in the same way (under irrigation and full sun). Adoption agroforestry shading in conilon coffee plantations can be useful to alleviate these climate negative effects, reducing the risks for coffee production in Espírito Santo. Agroforestry systems are able to reduce excess of radiation, mitigate temperature extremes and optimize moisture retention in soils. Technical-scientific knowledge on drought impacts (and their combination with other climate extremes effects) is an essential step in addressing the issue of vulnerability to drought in the state and can lead to mitigation-oriented drought management.

## Data Availability

Requests for materials should be addressed to: luan.venancio@ufv.br.
